# Dr. Courtault

**Published:** 1911-09-30

**Authors:** 


Dr. Courtault, author of a work on the Protection of Infants, a founder of the *Maison du Médecin*, and director of *Les Tablettes médicales mobiles de Paris* and of *La Médecine des Accidents du Travail*, has lost his life as the result of a collision between the packet-boat *L'Emir* and an English collier.

